# Associations of stunting in early childhood with cardiometabolic risk factors in adulthood

**DOI:** 10.1371/journal.pone.0192196

**Published:** 2018-04-11

**Authors:** Emanuella De Lucia Rolfe, Giovanny Vinícius Araújo de França, Carolina Avila Vianna, Denise P. Gigante, J. Jaime Miranda, John S. Yudkin, Bernardo Lessa Horta, Ken K. Ong

**Affiliations:** 1 Medical Research Council Epidemiology Unit, University of Cambridge School of Clinical Medicine, Institute of Metabolic Science, Cambridge, United Kingdom; 2 Post-graduate Program in Epidemiology, Federal University of Pelotas, Pelotas, Brazil; 3 Department of Medicine, School of Medicine, Universidad Peruana Cayetano Heredia, Lima, Peru; 4 University College London, London, United Kingdom; CUNY, UNITED STATES

## Abstract

Early life stunting may have long-term effects on body composition, resulting in obesity-related comorbidities. We tested the hypothesis that individuals stunted in early childhood may be at higher cardiometabolic risk later in adulthood. 1753 men and 1781 women participating in the 1982 Pelotas (Brazil) birth cohort study had measurements of anthropometry, body composition, lipids, glucose, blood pressure, and other cardiometabolic traits at age 30 years. Early stunting was defined as height-for-age Z-score at age 2 years below -2 against the World Health Organization growth standards. Linear regression models were performed controlling for sex, maternal race/ethnicity, family income at birth, and birthweight. Analyses were stratified by sex when p-interaction<0.05. Stunted individuals were shorter (β = -0.71 s.d.; 95% CI: -0.78 to -0.64), had lower BMI (β = -0.14 s.d.; 95%CI: -0.25 to -0.03), fat mass (β = -0.28 s.d.; 95%CI: -0.38 to -0.17), SAFT (β = -0.16 s.d.; 95%CI: -0.26 to -0.06), systolic (β = -0.12 s.d.; 95%CI: -0.21 to -0.02) and diastolic blood pressure (β = -0.11 s.d.; 95%CI: -0.22 to -0.01), and higher VFT/SAFT ratio (β = 0.15 s.d.; 95%CI: 0.06 to 0.24), in comparison with non-stunted individuals. In addition, early stunting was associated with lower fat free mass in both men (β = -0.39 s.d.; 95%CI: -0.47 to -0.31) and women (β = -0.37 s.d.; 95%CI: -0.46 to -0.29) after adjustment for potential confounders. Our results suggest that early stunting has implications on attained height, body composition and blood pressure. The apparent tendency of stunted individuals to accumulate less fat-free mass and subcutaneous fat might predispose them towards increased metabolic risks in later life.

## Introduction

The prevalence of obesity is rapidly increasing worldwide, particularly affecting low and middle-income countries, where this condition co-exists with undernutrition.[1,2] Childhood stunting is a significant global health issue, affecting 161 million children under 5 years of age in 2013.[3] It has a complex aetiology, involving household, environmental, socioeconomic and cultural factors.[4] Research has suggested that stunting or poor height gain in early childhood may be a critical factor in promoting later obesity and obesity-related comorbidities.[2,5,6]

Although the prevalence of stunting in the last decades has significantly decreased in middle income countries, it remains a global health priority due to its long-term consequences. In Brazil, national data show a reduction in the prevalence of childhood stunting from 37% in 1974–1975 to 7% in 2006–2007 [7], and in the city of Pelotas, a city in Southern Brazil, from 13.9% in 1982 to 5% in 2004.[8]

A systematic review by The Maternal and Child Undernutrition Study Group [9], which included cohort studies from five low- and middle-income countries (Brazil—The 1982 Pelotas birth cohort, Guatemala, India, the Philippines, and South Africa) has shown that poor growth early in life has longer-term consequences, such as shorter adult height, lower attained schooling, reduced adult income, and decreased offspring birthweight.

Currently, findings on the association of early stunting with later adiposity are controversial. In Guatemalan individuals, early stunting was associated with greater central adiposity after adjustment for overall fatness and potential confounders; however, studies in adults from New Delhi and Jamaican young adults failed to find evidence of an association between previous stunting and total or central adiposity. In addition, children who were undernourished in the first 2 years of life and gained weight rapidly later in childhood and adolescence were at a greater risk of obesity-related disease as they presented an unfavourable metabolic profile, including increased glucose concentrations, higher blood pressure and harmful lipid profiles.

Evidence from the prospective British 1946 Birth Cohort Study indicated a substantial effect of stunting in mid-childhood on higher mortality 30 to 60 years later.[10] The effects of stunting seemed also to influence the next generation, as women who were themselves stunted in childhood had a tendency to have stunted offspring.[11] Furthermore, early stunting seemed to be related to lower intelligence quotient, achieved schooling and income at 30 years of age.[12]

Previous analyses from the 1982 Pelotas (Brazil) birth cohort, however, failed to find evidence of associations of early stunting with metabolic syndrome components in young adults (mean age 22.8 years).[13] These inconsistencies may be partly explained by the different methods used to assess body composition. Most of these epidemiological studies relied on estimates of body fat and fat distribution from standard anthropometry.

Using data from the same cohort at age 30 years, we previously showed that men and women with early life stunting had lower subcutaneous abdominal fat thickness (SAFT), measured by the imaging technique ultrasound, but not visceral fat thickness (VFT) than other individuals.[14] In the present study, we now expanded on those analyses, focusing on the associations of early stunting with a wider range and more robust measurements of cardiometabolic traits in adulthood. We hypothesised that adults who were stunted in early childhood would have an unfavourable body composition and worse metabolic profile than those who did not experience stunting in early life.

## Materials and methods

### Study design and population

Details of the 1982 Pelotas Birth Cohort Study have been previously published elsewhere. [15–17] Briefly, it recruited 5914 live births in 1982 to mothers living in the urban part of Pelotas, a Southern Brazil city. The study participants have been followed up and examined at different times/phases. The most recent follow up was carried out in 2012–2013, which included a total of 3711 cohort members representing 68.1% of the original sample, including 325 members known to have died. In the current analysis, we included 3534 individuals, 1753 men and 1781 women, who had complete information on early stunting and selected cardiometabolic traits at age 30 years.[18]

In the early phases, verbal informed consent was obtained from the mothers, while in recent phases; written consent was obtained from each participant. The study was approved by the Ethical Committee of the Federal University of Pelotas and performed in accordance with the Declaration of Helsinki. The exclusion criteria for the ultrasound examination in the 2012–13 phase of the Pelotas study included pregnancy or probable pregnancy, as well as have given birth in the three months prior to the examination.

### Study measurements

#### Anthropometry and body composition

Measurements were performed by trained staff following standard protocols. Adults’ weight was recorded to the nearest 0.1 kg using calibrated electronic scales (TANITA BC-418 MA; Tanita, Tokyo, Japan). Supine length and height was measured to the nearest 0.1cm in 1984 and 2013, respectively, using boards manufactured locally according to international specifications (AHRTAG; Healthlink Worldwide, London, UK). Adults’ height was measured to the nearest 0.1cm using a wall-mounted stadiometer (SECA 240; Seca, Birmingham, UK). Early stunting was defined as height-for-age Z-score below -2 at age 2 years using the World Health Organization (WHO) criteria [19]. Body mass index (BMI; in kg/m^2^) was calculated as weight divided by square height.Fat mass (kg) fat free mass (kg) were derived using the air-displacement plethysmography (Bod Pod^®^, Cosmed, Concord, CA USA). Body density was firstly calculated as mass/body volume and body fat percentage was estimated using the Siri equation.[20]

A Toshiba Xario (Toshiba Medical Systems Corporation, Tochigi-ken, Japan) ultrasound machine with a 3.5-MHz convex transducer was used to derive visceral and subcutaneous (total, deep and superficial) abdominal fat thicknesses. VFT was estimated by the distance between the peritoneum and the lumbar spine and SAFT was defined as the distance between the posterior line of dermis to the linea alba. Both measurements were obtained at the intersection between the xyphoid line and the waist circumference. The relative intra-observer technical error of measurement for the visceral thickness was 4.1% and 3.4% for subcutaneous fat thickness, whereas the relative inter-observer technical error of measurement was 3.1% for both measurements.

#### Biochemistry and clinical measurements

Non-fasting blood samples were collected by venous puncture. Whole blood samples were spotted onto filter paper (Whatman 903; Maidstone, UK), air-dried, and stored in sealed envelopes at ambient temperature. Random glucose was measured by an automatic enzymatic colorimetric method. Glycated haemoglobin (HbA1c) was measured by the Variant (Bio-Rad, Hercules, CA) ion-exchange high-performance liquid chromatography (HPLC) method. High sensitive C reactive protein was analysed using immune-turbidimetry test with intra-inter assay coefficient of variation of 4.2%.

Blood lipids (total cholesterol, high and low-density lipoprotein and triglycerides) were measured by standard enzymatic methods. All the above analyses were run in an automated Mindray BS 380 Chemistry Analyser (Shenzhen Mindray Bio-Medical Electronics Co., Ltd, China).

Carotid intima-media thickness (CIMT) was measured with a Toshiba Xario (Toshiba Medical Systems Corp) ultrasound machine with a 3.5-MHz linear transducer. CIMT was measured at the posterior wall of the right and left common carotid arteries in longitudinal planes, through an image of a 10-mm long section proximal to the carotid bulb. The image were analysed using the Carotid Analyser for Research (Medical Imaging Applications, MIA-LLC), which automatically calculated the mean value of 90 measurements (frames) taken [21].

Arterial blood pressure was measured twice (at the beginning and end of the examination) with an automated blood pressure monitor (Omron HEM-705CPINT) with the participant rested and seated for at least five minutes.

#### Other covariates

Information on *a priori* potential confounders was collected. Family income in 1982 was calculated as the sum of the monthly incomes of all working persons living in the household, expressed in multiples of the minimum wage (≤1/1.1-3/3.1–6; >6). Maternal self-reported skin colour was collected as a marker of race/ethnicity and categorized into two groups (white/non-white). Birthweight was measured to the nearest 10g using calibrated paediatric scales (Filizola, São Paulo, Brazil) by trained anthropometrists following standard protocols.

### Statistical analysis

Statistical analyses were performed with Stata (version 13; StataCorp, College Station, TX). Descriptive data are presented as mean ± standard deviation (s.d.) or median and interquartile range. Inter-correlations between the independent variables were assessed by Spearman’s correlation. All outcomes were log-transformed to achieve normal distributions, and were posteriorly standardized to allow direct comparisons of the regression coefficients. Therefore, the Beta (β) coefficients indicate the standardised difference in the outcomes comparing stunted versus non-stunted individuals.

Inter-correlations between the independent variables were assessed by Spearman’s correlation. Co-linearity between parameters was indicated by a variance inflation factor (VIF) > 5.

Linear regression models were derived to analyse the relationship between stunting at age 2 years (exposure) and the various body composition and the metabolic traits variables (outcomes). We initially tested whether sex modified the association of stunting at age 2 years with each outcome at age 30 years, and presented the analyses stratified by sex when p-interaction<0.05. For each outcome, we ran crude models (including only the outcome and stunting at age 2 years), as well as models adjusted for potential confounders measured at birth (sex, maternal race/ethnicity, family income at birth, and birthweight). We also assessed the association between stunting at age 2 years and VFT adjusting for potential confounders and current BMI, for comparative purposes. Additional inverse-probability weighted models were applied, which account for possible selection bias in cohort follow-up.

We applied inverse-probability weighting (IPW) [22] to take into account selection bias by using the teffects ipw command in Stata. For comparative purposes, IPW models were adjusted separately for each outcome, including the same potential confounders used in the linear regression models. The 95% confidence intervals (CI) were estimated using the jackknife method. A 5% significance level was applied.

## Results

Characteristics of the study population are summarised in Table 1. The mean age of the participants was 30.20years (s.d. = 0.34). Men had greater length at 2 years, attained height at 30 years of age, weight, BMI, fat-free mass, VFT, systolic and diastolic blood pressure, random glucose level, total and low-density lipoprotein (LDL), and triglycerides, but lower fat mass, subcutaneous abdominal fat, C-reactive protein, and high-density lipoproteins (HDL) than women at age 30 years.

**Table 1:** Characteristics of the study sample.

Traits	Men	Women	p-value§
	*n* = 1772	*n* = 1853	
Length at 2 years (cm)	-0.74 (-1.62)	-0.58 (1.54)	<0.0001
Height (cm) at 30 years	174.50 (8.80)	161.30 (8.30)	<0.0001
Weight (kg) at 30 years	80.20 (20.20)	65.90 (20.00)	0.0001
BMI (kg/m2) at 30 years	26.33 (5.82)	25.32 (7.19)	<0.0001
Fat mass (kg) at 30 years	19.77 (14.28)	24.57 (15.06)	0.0001
Fat mass (%) at 30 years	25.10 (12.90)	37.30 (12.00)	<0.0001
Fat-free (kg) at 30 years	60.51 (10.18)	41.82 (6.95)	<0.0001
VFT (cm) at 30 years	6.64 (2.57)	4.63 (1.98)	<0.0001
Total SAFT (cm) at 30 years	1.76 (1.29)	2.42 (1.60)	<0.0001
Ratio VFT/SAFT	4.61±3.15	2.31±1.39	<0.0001
Mean carotid intima media thickness (cm)	0.59±0.02	0.58±0.02	<0.0001
Systolic blood pressure (mmHg) at 30 years	127.00 (16.00)	113.50 (14.50)	0.0001
Diastolic blood pressure (mmHg) at 30 years	76.50 (12.50)	73.00 (11.00)	0.0001
Glycated haemoglobin (HbA1c)* at 30 years	5.10 (0.40)	5.10 (0.40)	0.01
C-reactive protein (mg/L) at 30 years	2.79±4.36	5.05±6.16	<0.0001
Random glucose (mg/dL) at 30 years	92.52±30.30	86.65±20.50	<0.0001
Total cholesterol (mg/dL) at 30 years	189.00 (51.00)	186.00 (48.00)	0.02
HDL cholesterol (mg/dL) at 30 years	52.00 (16.00)	63.00 (19.00)	<0.0001
LDL cholesterol (mg/dL) at 30 years	110.00 (40.00)	103.00 (36.00)	0.0001
Triglycerides (mg/dL) at 30 years	140.53±129.12	102.42±61.25	<0.0001

Data are means ± s.d. or median (interquartile range).

^§^ Mann-Whitney-Wilcoxon test

VFT—visceral fat by ultrasound; SAFT—subcutaneous abdominal fat by ultrasound;

*HbA1c shown as percentage of total haemoglobin

Stunting at age 2 years was more prevalent in men than in women (16.3% and 11.6%, respectively). It was higher, almost double, amongst black individuals than whites, and it was much higher, six to ten times more, among those in the poorest families relative to those in the highest income category (Table 2).

**Table 2:** Stunting at age 2 years by covariates.

Variables	Men					Women				
	*n*	% of stunting at 2 years	95% CI		p-value	*n*	% of stunting at 2 years	95% CI		p-value
Overall	3,037	16.3	14.9	17.8	-	2,876	11.6	10.4	12.9	-
*Maternal race/ethnicity*					*<0*.*001*					*<0*.*001*
White	2,479	14.6	13.1	16.2		2,371	9.5	8.3	10.8	
Non-white	556	23.7	20.0	27.8		504	21.8	18.1	26.0	
*Family income at birth (in minimum wages)*					*<0*.*001*					*<0*.*001*
< = 1	666	29.9	26.1	34.1		622	24.6	21.0	28.7	
1.1–3	1,463	16.7	14.7	18.9		1,325	10.8	9.2	12.8	
3.1–6	544	7.5	5.5	10.2		547	5.8	4.0	8.4	
>6	351	5.2	3.2	8.5		366	2.4	1.1	4.9	

The correlations between the independent variables are shown in Table 3.

**Table 3:** Correlation (Spearman’s ρ) between the independent variables.

Variables	Attained height	BMI	Fat mass	Fat free mass	% body fat	VFT	Total SAFT	VFT/ SAFT ratio	Mean CMIT	Systolic blood pressure	Diastolic blood pressure	Glycated haemoglobin	C-reactive protein	Random glucose	Total cholesterol	HDL cholesterol	LDL cholesterol
**BMI**	-0.0162	1															
**Fat mass**	**0.1571**	**0.8681**	1														
**Fat free mass**	**0.3012**	**0.3533**	**0.0416**	1													
**VFT**	**-0.0457**	**0.5356**	**0.3464**	**0.5955**	**0.4902**	1											
**Total SAFT**	**0.0901**	**0.7296**	**0.8415**	-0.0336	**0.7715**	**0.1598**	1										
**VFT/SAFT ratio**	**-0.1067**	**-0.3215**	**-0.5338**	**0.4013**	**-0.3865**	**0.4492**	**-0.783**	1									
**Mean CMIT**	**0.0471**	**0.3371**	**0.2391**	**0.3052**	**0.2547**	**0.3436**	**0.1613**	**0.0699**	1								
**Systolic BP**	**0.097**	**0.2943**	**0.2729**	**0.1451**	**0.2442**	**0.2027**	**0.2294**	**-0.0731**	**0.1646**	1							
**Diastolic BP**	0.0275	**0.3431**	**0.3612**	**0.087**	**0.3637**	**0.2083**	**0.3122**	**-0.1369**	**0.1402**	**0.7429**	1						
**Glycated haemoglobin**	0.0024	**0.1049**	**0.082**	**0.0741**	**0.0899**	**0.093**	**0.0612**	0.0027	**0.0674**	**0.0846**	**0.065**	1					
**C-reactive protein**	0.0016	**0.3638**	**0.4611**	**-0.1595**	**0.4005**	**0.1053**	**0.3975**	**-0.2859**	**0.0827**	**0.186**	**0.2291**	**0.0737**	1				
**Random glucose**	-0.0253	**0.1731**	**0.1262**	**0.1765**	**0.1756**	**0.2669**	**0.0844**	**0.088**	**0.1076**	**0.1256**	**0.1282**	0.0208	**0.0375**	1			
**Total cholesterol**	**-0.0386**	**0.2021**	**0.223**	0.0099	**0.2468**	**0.1642**	**0.1714**	**-0.063**	**0.1381**	**0.1358**	**0.1755**	0.015	**0.1363**	**0.184**	1		
**HDL cholesterol**	-0.0014	**-0.2459**	**-0.2044**	**-0.1111**	**-0.1896**	**-0.2164**	**-0.1675**	0.0149	**-0.1426**	-0.032	**-0.0424**	**-0.0599**	**-0.0619**	**-0.0553**	**0.321**	1	
**LDL cholesterol**	**-0.0648**	**0.2348**	**0.2395**	0.0241	**0.2662**	**0.1817**	**0.1939**	**-0.0722**	**0.1711**	**0.1207**	**0.1609**	0.0134	**0.1297**	**0.1082**	**0.8837**	**0.085**	1
**Triglycerides**	-0.0119	**0.3418**	**0.2905**	**0.239**	**0.3492**	**0.4019**	**0.2194**	**0.042**	**0.1906**	**0.1508**	**0.1823**	**0.0623**	**0.1332**	**0.3062**	**0.4511**	**-0.2271**	**0.3083**

VFT—visceral fat by ultrasound

SAFT—subcutaneous abdominal fat by ultrasound

BP—Blood Pressure

Bold values signifies p<0.05.

Associations of stunting at age 2 years with cardiometabolic traits at age 30 years are shown in Table 4. In the crude models, stunting at 2 years was associated with lower adult height, current BMI, fat-mass, and total SAFT, and with higher VFT, VFT/SAFT ratio and random glucose concentration. After adjustment for potential confounders, stunted individuals were shorter (β = -0.71 s.d.; p<0.001); had lower BMI (β = -0.14 s.d.; p = 0.01), fat mass (β = -0.28 s.d.; p<0.001), SAFT (β = -0.16 s.d.; p = 0.002), systolic blood pressure (β = -0.12 s.d.; p = 0.01), and diastolic blood pressure (β = -0.11 s.d.; p = 0.04); and higher VFT/SAFT ratio (β = 0.15 s.d.; p = 0.001), in comparison with non-stunted individuals. Higher visceral fat thickness (β = 0.08 s.d.; p = 0.04) in stunted individuals at 2 years only appeared after adjustment for current BMI.

**Table 4:** Associations of stunting at age 2 years with cardiometabolic traits at age 30 years.

Traits (s.d. ln)	Crude					Adjusted*				
	N	Beta	95%CI		p-value	N	Beta	95%CI		p-value
Height	3309	-0.72	-0.82	-0.62	**<0.001**	3294	-0.71	-0.78	-0.64	**<0.001**
BMI	3256	-0.17	-0.28	-0.07	**0.001**	3242	-0.14	-0.25	-0.03	**0.01**
Fat mass	3231	-0.40	-0.50	-0.30	**<0.001**	3217	-0.28	-0.38	-0.17	**<0.001**
VFT	3203	0.11	0.00	0.21	**0.04**	3189	0.00	-0.09	0.09	0.99
Total SAFT	3221	-0.25	-0.35	-0.15	**<0.001**	3207	-0.16	-0.26	-0.06	**0.002**
VFT/SAFT ratio	3203	0.28	0.18	0.39	**<0.001**	3189	0.15	0.06	0.24	**0.001**
Mean CMIT	2922	-0.02	-0.13	0.08	0.65	2909	-0.06	-0.18	0.05	0.27
Systolic blood pressure	3319	0.01	-0.09	0.12	0.80	3304	-0.12	-0.21	-0.02	**0.01**
Diastolic blood pressure	3319	-0.04	-0.14	0.06	0.45	3304	-0.11	-0.22	-0.01	**0.04**
C-reactive protein	3242	-0.06	-0.17	0.04	0.22	3228	0.00	-0.11	0.10	0.98
Random glucose	3242	0.12	0.02	0.23	**0.02**	3228	0.08	-0.03	0.19	0.15
LDL cholesterol	3242	-0.03	-0.13	0.07	0.55	3228	-0.06	-0.17	0.05	0.27
Triglycerides	3242	-0.07	-0.18	0.03	0.16	3228	-0.08	-0.19	0.02	0.11

*Adjusted for sex, family income at birth, maternal race/ethnicity, and birthweight.

BMI—body mass index; VFT—visceral fat thickness; SAFT—subcutaneous abdominal fat thickness; CMIT—carotid intima-media thickness.

Bold values signifies p<0.05.

Table 5 presents sex-specific analyses performed for those outcomes in which sex modification was identified (p-interaction<0.05). After adjustment for potential confounders, early stunting was associated with lower fat free mass in both men (β = -0.39 s.d.; p<0.001) and women (β = -0.37 s.d.; p<0.001). Additional inverse-probability weighted models showed essentially similar associations (S1 and S2 Tables).

**Table 5:** Associations of stunting at age 2 years with glycated haemglobin, total cholesterol and HDL cholesterol, and fat free mass at age 30 years by sex.

Traits (s.d. ln)	Crude					Adjusted*				
	N	Beta	95%CI		p-value	N	Beta	95%CI		p-value
*Males*										
Fat free mass	1587	-0.50	-0.58	-0.42	**<0.001**	1583	-0.39	-0.47	-0.31	**<0.001**
Glycated haemoglobin	1591	0.12	-0.02	0.26	0.09	1587	0.09	-0.06	0.23	0.26
Total cholesterol	1590	0.04	-0.10	0.19	0.56	1586	0.04	-0.11	0.20	0.57
HDL cholesterol	1590	0.14	0.01	0.27	**0.04**	1586	0.12	-0.02	0.26	0.08
*Females*										
Fat free mass	1644	-0.41	-0.50	-0.32	**<0.001**	1634	-0.37	-0.46	-0.29	**<0.001**
Glycated haemoglobin	1652	-0.13	-0.28	0.03	0.11	1642	-0.15	-0.31	0.01	0.07
Total cholesterol	1652	-0.19	-0.34	-0.04	**0.01**	1642	-0.16	-0.31	0.00	0.05
HDL cholesterol	1652	-0.24	-0.38	-0.09	**0.001**	1642	-0.13	-0.27	0.02	0.09

*Adjusted for family income at birth, maternal race/ethnicity, and birthweight.

Bold values signifies p<0.05.

## Discussion

Our results from this large, long-running, middle-income country birth cohort study suggest that stunting at age 2 years has implications on attained height, body composition and blood pressure. Consistently with the current literature, we observed that men and women with early stunting were shorter as adults than non-stunted individuals.

Central obesity has been reported to be greater in stunted individuals who were undernourished in childhood, born in Jamaica in the 1970, and in 1986.[23–25] However, most of these studies relied on anthropometric indicators of body fat, such as waist and hip circumferences and skinfold thickness, rather than directly assessed specific fat compartments, such as visceral and subcutaneous fat, given that the distribution of these fat compartments may have different metabolic consequences. Visceral fat has been associated with a low-grade inflammation due to the increased secretion of numerous pro-inflammatory cytokines, which may play an important role in many diseases, promoting angiogenesis, inflammation, cell proliferation, and insulin resistance.[26–28] Although some studies have shown that subcutaneous fat may have an independent antiatherogenic effect, others have suggested that it might be a significant predictor of adverse metabolic consequences in some populations.[29,30] In our study, we found an association between early stunting and higher visceral fat accumulation in adulthood only after adjustments for current BMI. In addition, our findings also suggest that early stunting may decrease lean mass, subcutaneous abdominal fat accumulation, as well as total body fat mass.

Childhood stunting may lead to less lean mass as individuals who are undernourished tend to use up protein stores in the muscle as a source of energy as a consequence of inadequate energy intake[31]. Muscle tissue catabolism is significantly greater during protein–energy malnutrition and results in the reduction in the muscle mass in those individuals [32]. The loss of subcutaneous fat may also be a result of insufficient food intake that is deficient of calories and protein. Reduction in subcutaneous fat is often seen in children with marasmus [33,34]. An important characteristic of marasmus is growth failure, which results in those children not reaching their full potential height (stunted). While, children with kwashiorkor retain subcutaneous fat as calorie intake is adequate but are protein deficient [33,34].

This body composition profile resulting from stunting might be harmful in relation to metabolic disorders. Lean mass is the largest insulin-sensitive tissue in the body and plays a pivotal role in the maintenance of glucose metabolism.[35,36] Loss or reduced lean mass may therefore play a fundamental role in altering glucose homeostasis including metabolic dysregulation of insulin stimulated glucose uptake and may contribute to the development of metabolic disorders later in life.[37] Low lean mass has also been found to be the strongest risk factors for metabolic syndrome in adults, independent of abdominal fat.[38] The mechanisms contributing to reduced insulin stimulated glucose uptake, impaired insulin signalling and action on lean mass are not fully understood. Increased accumulation of adipose tissue and intramuscular fat, dysregulated production of inflammatory adipokines, increased renin-angiotensin-aldosterone system activity, and decreased mitochondrial oxidative phosphorylation flux in the muscle have been suggested to play a significant role in the development of insulin resistance in lean mass.[37]

In addition, reduced subcutaneous fat may contribute to fat accumulation in other tissues and organs. Failure of fat cell formation in the subcutaneous fat compartment as body fat increases, might lead to storage of fat in tissues such as visceral adipose tissue, pancreas, muscle and liver.[39] This excess storage might contribute to the unfavourable distribution of abdominal fat and hepatic steatosis, as well as impaired insulin action resulting in insulin resistance, glucose intolerance and diabetes.[40]

Our results on cardiovascular and metabolic traits were not consistent across sex. Men who were stunted had higher HDL cholesterol levels, while stunted women had lower total cholesterol than the non-stunted ones. Previous analyses from the 1982 Pelotas (Brazil) birth cohort assessing associations with early stunting, defined using the 2006 WHO Child Growth Standards for height-for-age z score, showed mixed results, Nazmi et al. [41] found that men who were stunted at age 2 years and had abdominal obesity at age 23y had higher C-reactive protein levels, suggesting an increased risk for chronic diseases. Grillo et al. [13], studying metabolic syndrome components at adulthood (mean age 22.8 years), found that men and women who were stunted at age 2 years had lower triglycerides and women had lower HDL-cholesterol levels; however, these associations disappeared after adjustment for confounding variables. Buffarini et al.[42], using data from the 1982 and 1993 Pelotas (Brazil) birth cohorts, reported that those individuals who were stunted at age 1 had lower glycated haemoglobin in adolescents aged 18y, but not in adults aged 30 years. These discrepancies may be partly explained by excluding potential confounders like birth weight and or including mediating variables such as attained height and BMI to the analysis. These adjustments might potentially introduce bias because of inappropriate controlling.

We acknowledge some limitations of our study. Lipids and glucose levels were measured from non-fasting blood samples is our study; while HDL cholesterol is independent of fasting time [43], triglycerides are influenced by time of the day and fasting time.[44] However, new evidence suggests that the impact of fasting on lipids is modest [45]. We relied on ultrasound measurements of VFT and SAFT as proxies for these abdominal fat compartments. This technique has been found to correlate strongly with magnetic resonance imaging estimates of abdominal fat compartments in a variety of settings and populations using the same protocol, which was rigorously quality controlled.[46–48] We cannot rule out residual confounding, as we cannot exclude unmeasured genetic and environmental factors that may contribute to those associations.

To our knowledge, this is one of the largest reported samples with abdominal ultrasound measurements, and by far the largest from a low- or middle-income setting, which addressed the associations of early stunting with several cardiometabolic traits in adulthood. The prospective design of our study made it less susceptible to recall bias, as potential confounding variables were measured soon after delivery, and the higher follow-up rate of 68.1% at age 30 years is likely to reduce the chance of selection bias. The use of objective measurements/indicators of body composition is also a strength of this analysis.

In conclusion, our findings indicate that adults who had early childhood stunting may tend to accumulate less lean mass and subcutaneous fat, and may have a tendency to have a profile with higher visceral fat. This body composition profile, characterized by a preference to accumulate visceral fat rather than subcutaneous fat, might be deleterious to their future metabolic health. The current and previous reported findings derived from this cohort do not yet show evidence for increased biochemical markers of metabolic disease later into early adulthood, possibly because the cohort is still young to present marked alterations in the evaluated traits. Our findings of a worse adiposity profile associated with stunting in early childhood do not preclude, however, that further developments into negative cardiometabolic profiles may occur, thus warranting longer-term evaluations.

## Supporting information

S1 Table Associations of stunting at age 2y with cardiometabolic traits at age 30y. (DOCX)

S2 Table Associations of stunting at age 2y with glycated haemglobin, total cholesterol and HDL cholesterol at age 30y according to sex. (DOCX)
