# Supplementary material for: Associations of stunting in early childhood with cardiometabolic risk factors in adulthood
Source: PLoS One. 2018 Apr 11;13(4):e0192196. doi: 10.1371/journal.pone.0192196 (PMC5894958; doi:10.1371/journal.pone.0192196)
Supplement: S1 Table — (DOCX) [file pone.0192196.s001.docx]

**SUPPORTING INFORMATION**

**S1 Table**. Associations of stunting at age 2y with cardiometabolic traits at age 30y

| **Variables (s.d. ln)** | **Adjusted* linear regression** | | | | |  | **Adjusted* inverse probability weighting**** | | | |
| --- | --- | --- | --- | --- | --- | --- | --- | --- | --- | --- |
|  | **N** | **Beta** | **95%CI** | | **p-value** |  | **Beta** | **95%CI** | | **p-value** |
| Height | 3294 | -0.71 | -0.78 | -0.64 | <0.001 |  | -0.79 | -0.90 | -0.68 | <0.001 |
| BMI | 3242 | -0.14 | -0.25 | -0.03 | 0.010 |  | -0.15 | -0.33 | 0.03 | 0.111 |
| Fat mass | 3217 | -0.28 | -0.38 | -0.17 | <0.001 |  | -0.27 | -0.42 | -0.12 | <0.001 |
| VFT | 3189 | 0.00 | -0.09 | 0.09 | 0.998 |  | 0.09 | -0.07 | 0.24 | 0.262 |
| Total SAFT | 3207 | -0.16 | -0.26 | -0.06 | 0.002 |  | -0.12 | -0.25 | 0.01 | 0.069 |
| VFT/SAFT ratio | 3189 | 0.15 | 0.06 | 0.24 | 0.001 |  | 0.15 | 0.04 | 0.27 | 0.010 |
| Mean CMIT | 2909 | -0.06 | -0.18 | 0.05 | 0.271 |  | -0.06 | -0.19 | 0.08 | 0.405 |
| Systolic blood pressure | 3304 | -0.12 | -0.21 | -0.02 | 0.015 |  | -0.09 | -0.22 | 0.05 | 0.214 |
| Diastolic blood pressure | 3304 | -0.11 | -0.22 | -0.01 | 0.038 |  | -0.08 | -0.23 | 0.08 | 0.351 |
| C-reactive protein | 3228 | 0.00 | -0.11 | 0.10 | 0.982 |  | 0.00 | -0.15 | 0.14 | 0.957 |
| Random glucose | 3228 | 0.08 | -0.03 | 0.19 | 0.155 |  | 0.11 | -0.01 | 0.23 | 0.083 |
| LDL cholesterol | 3228 | -0.06 | -0.17 | 0.05 | 0.270 |  | -0.10 | -0.31 | 0.10 | 0.319 |
| Triglycerides | 3228 | -0.08 | -0.19 | 0.02 | 0.114 |  | -0.02 | -0.16 | 0.12 | 0.781 |

*Adjusted for sex, family income at birth, maternal self-reported race/ethnicity, and birthweight.

**Standard errors derived by jackknife procedure.
