# Supplementary material for: Associations of stunting in early childhood with cardiometabolic risk factors in adulthood
Source: PLoS One. 2018 Apr 11;13(4):e0192196. doi: 10.1371/journal.pone.0192196 (PMC5894958; doi:10.1371/journal.pone.0192196)
Supplement: S2 Table — (DOCX) [file pone.0192196.s002.docx]

**SUPPORTING INFORMATION**

**S2 Table.** Associations of stunting at age 2y with glycated haemglobin, total cholesterol and HDL cholesterol at age 30y according to sex

| **Variables (s.d. ln)** | **Adjusted* linear regression** | | | | |  | **Adjusted* inverse probability weighting**** | | | |
| --- | --- | --- | --- | --- | --- | --- | --- | --- | --- | --- |
|  | **N** | **Beta** | **95%CI** | | **p-value** |  | **Beta** | **95%CI** | | **p-value** |
| Males |  |  |  |  |  |  |  |  |  |  |
| Fat free mass | 1583 | -0.39 | -0.47 | -0.31 | **<0.001** |  | -0.48 | -0.58 | -0.39 | **<0.001** |
| Glycated  haemoglobin | 1587 | 0.09 | -0.06 | 0.23 | 0.257 |  | 0.15 | -0.10 | 0.40 | 0.234 |
| Total cholesterol | 1586 | 0.04 | -0.11 | 0.20 | 0.573 |  | 0.11 | -0.07 | 0.28 | 0.243 |
| HDL cholesterol | 1586 | 0.12 | -0.02 | 0.26 | 0.083 |  | 0.18 | 0.04 | 0.33 | **0.013** |
| Females |  |  |  |  |  |  |  |  |  |  |
| Fat free mass | 1634 | -0.37 | -0.46 | -0.29 | **<0.001** |  | -0.37 | -0.49 | -0.25 | **<0.001** |
| Glycated  haemoglobin | 1642 | -0.15 | -0.31 | 0.01 | 0.070 |  | -0.30 | -0.62 | 0.03 | 0.079 |
| Total cholesterol | 1642 | -0.16 | -0.31 | 0.00 | **0.048** |  | -0.14 | -0.39 | 0.10 | 0.251 |
| HDL cholesterol | 1642 | -0.13 | -0.27 | 0.02 | 0.094 |  | -0.15 | -0.47 | 0.16 | 0.342 |

*Adjusted for family income at birth, maternal self-reported race/ethnicity, and birthweight.

**Standard errors derived by jackknife procedure.
